# A 20-year investigation of declining leatherback hatching success: implications of climate variation

**DOI:** 10.1098/rsos.170196

**Published:** 2017-10-04

**Authors:** Anthony R. Rafferty, Christopher P. Johnstone, Jeanne A. Garner, Richard D. Reina

**Affiliations:** 1 School of Biological Sciences, Monash University, Melbourne, Australia; 2 West Indies Marine Animal Research and Conservation Service, Frederiksted, St Croix, US Virgin Islands

**Keywords:** *Dermochelys coriacea*, egg death, nesting, precipitation, St Croix Virgin Islands, temperature

## Abstract

Unprecedented increases in air temperature and erratic precipitation patterns are predicted throughout the twenty-first century as a result of climate change. A recent global analysis of leatherback turtle hatchling output predicts that the nesting site at Sandy Point National Wildlife Refuge (SPNWR) will experience the most significant regional climate alterations. We aimed to identify how local air temperatures and precipitation patterns influenced within-nest mortality and overall hatchling output at this site between 1990 and 2010. We show that while the greatest mortality occurred during the latest stages of development (stage three), the rate of embryo mortality was highest during the initial stages (stage zero) of development (approx. 3.8 embryos per day per clutch). Increased mortality at stage three was associated with decreased precipitation and increased temperature during this developmental period, whereas precipitation prior to, and during stage zero had the greatest influence on early mortality. There was a significant decline in overall hatching success (falling from 74% to 55%) and emergence rate (calculated from the number of hatchlings that emerged from the nest as a percentage of hatched eggs) which fell from 96% to 91%. However, there was no trend observed in local temperature or precipitation during this timeframe, and neither variable was related to hatching success or emergence rate. In conclusion, our findings suggest that despite influencing within-nest mortality, climatic variability does not account for the overall decline in hatchling output at SPNWR from 1990 to 2010. Further research is therefore needed to elicit the reasons for this decline.

## 1. Introduction

Projections in the fifth assessment report by the Intergovernmental Panel on Climate Change (IPCC) predict an ongoing increase in global air temperature, more erratic precipitation patterns, continued sea-level rise and ocean acidification [1]. Collectively, changes to both terrestrial and marine environments have the potential to significantly impact marine turtle populations because they occupy both habitats during ontogeny [2,3]. Research into such impacts on leatherback turtle populations has intensified during the last decade following identification of the precipitous and critical decline of this species in the Pacific Ocean [4]. In an attempt to understand the possible causes for such drastic declines in this region, studies began focusing on the effects of climate variability on juvenile and adult turtles in the open ocean [5,6], and on developmental success of embryos on the nesting beach [7].

Research to date shows that decreased oceanic productivity in foraging and migration areas, caused by climatic variability, alters remigration intervals and negatively affects egg production in leatherback turtles [5,6,8]. Additionally, increased air temperature and decreased precipitation on nesting beaches is detrimental because it reduces hatching and emergence success [9,10]. The latest IPCC report details the successive decadal increase in air temperature over the last three decades, which is greater than any foregoing decadal increase since 1850 [1]. Rising temperatures and continued beach warming are predicted to reduce the Costa Rican leatherback population by approximately 7% per decade during the twenty-first century [10].

Temperature and precipitation patterns influence the timing of the sea turtle nesting season [11] and alter the thermal and hydric nest environment, which largely directs embryonic development and determines hatchling sex during incubation [12–14]. Throughout incubation, the developing embryo exchanges heat, carbon dioxide, oxygen and water with its surrounding environment via a permeable eggshell [15,16]. Nest environmental variables are closely interlinked and an extreme in any one of them can have detrimental effects on development, which is of primary concern given rising global temperatures and changing precipitation patterns.

The thermal environment of the nest dictates the rate of embryonic development and successful growth only occurs within a tolerance range between 25°C and 35°C [15]. Temperatures outside this range cause embryo mortality, which has also been documented in pre-emergent marine turtle hatchlings when nest sand temperatures exceed 33°C [17]. Furthermore, hatchling sex is also dictated by incubation temperature (temperature dependent sex determination; TSD), which may give rise to sex bias and the generation of more females at higher temperatures. The pivotal temperature at which marine turtle sex is determined is generally approximately 29°C and appears to be conserved at both a species and population level [18]. The threat imposed by high nest temperatures is somewhat offset by precipitation, which, in moderation, decreases temperature within the nest [12,19,20]. However, excessive precipitation can decrease gas exchange, causing egg and hatchling mortality [21,22].

Despite the profound impact that altered incubation conditions have on embryo development, limited research exists that looks at the bearing that climate variability can have on offspring production [23,24]. This is particularly pertinent in the case of the leatherback turtle, which is anomalous among the seven species of marine turtle for having an extremely low average hatching success of approximately 50% [25,26]. A high rate of early embryo mortality following oviposition is the primary determinant of this low hatching success, rather than infertility [25]. Differing reproductive success among females has been linked to early embryonic mortality, but much variability still remains to be explained and may be linked with developmental sensitivity to environmental conditions [27].

The impact of climate variability on hatchling production can only be fully understood from assessing long-term trends in data [28]. Marine turtles live and nest for many years, and evaluating reproductive output in a given year does not accurately reflect a lifetime of reproductive investment. On a year to year basis, factors dictating the nest environment (e.g. nest position and time of nesting) are very important, but on a larger time scale, inter-annual variation may prove to be a greater determinant of overall success. Furthermore, the potentially feminizing effects that increased temperature may have on both a rookery and population level can only be determined from examining sex ratios over time, because ratios may vary among years and nesting beaches within a population [28]. Failure to gain an in-depth knowledge of climate change impacts on a population level is currently constraining effective management [29].

A recent global analysis of leatherback turtle hatchling output predicted that the nesting site at Sandy Point National Wildlife Refuge (SPNWR) on St Croix, US Virgin Islands, will experience the most major future regional climate alterations [30]. We aimed to identify how historical fluctuations in local ambient air temperatures and precipitation patterns influenced within nest mortality and overall hatchling output at this site. To ensure a long-term, robust dataset we used 20 years of nesting and environmental data (1990–2010), collected at Sandy Point nesting beach, which is the site of the world's longest running saturation-tagging programme for leatherback turtles.

## 2. Material and methods

### 2.1. Study site and data collection

Data for this study were collected at SPNWR (17°41 N, 64°54 W), during the leatherback turtle nesting season (April–August) each year between 1990 and 2010. Nesting on SPNWR occurs within a 3.0 km range spanning both a north-facing open shore and a southeast facing heavily vegetated shore prone to large amounts of deposition [31]. An estimated abundance of approximately 695 nests per annum are being laid at SPNWR, with a generally increasing trend in the nesting population [32]. Data collection methods at this site have previously been described [33]. Nightly beach patrols were conducted during the monitoring period on an hourly basis between 20.00 and 05.00 [34] to locate and identify female turtles during nesting. Remigrant females (having nested at least once in a previous season) were identified by flipper and/or passive integrated transponder tags previously applied. Females without tags were recorded and tagged for identification in subsequent years. Successful nesting events were recorded and in some instances, nests laid in an erosion-prone location or below the high-tide mark were relocated to a hatchery or stable area of the beach. Nest location (*in situ* and relocated) was triangulated for later identification and excavation following hatchling emergence. Data collection was in accordance with appropriate ethical approval and licensing.

### 2.2. Nest excavation and measurement of embryo mortality

Nests were excavated approximately 24 h after initial hatchling emergence and the developmental success of each clutch was determined. The numbers of hatched shells, unhatched eggs and dead hatchlings in each nest were counted so that the hatching success (hatched egg shells/total eggs) and emergence rate (hatchlings emerged from nest/total hatched eggs) of each clutch could be determined. Eggs that died of extrinsic causes (e.g. invasion by plant roots or predation) were excluded. Unhatched eggs were opened and the developmental stage at death was determined based on the presence/absence of anatomical features. For this study, embryos were assigned to one of four ‘field stages’ (ranked zero–three) based on the method described by Leslie *et al*. [35], which corresponds to Miller's [36] developmental chronology for sea turtles, and is consistent with previous research (e.g. [16,27]). Field stage zero included eggs up to approximately 4 days post-oviposition in which no embryo or blood vessels were visible; field stage one included eggs estimated to be between 4 and 9 days post-oviposition, in which visible blood vessels and an unpigmented embryo were present; field stage two included eggs estimated to be between 9 and 24 days post-oviposition containing embryos with pigmented eyes; and field stage three included eggs with fully pigmented embryos estimated to be between 24 days and full term (approx. 60+ days). Dead hatchlings that failed to emerge from the nest were also included in this latter field stage so that a clear and functional explanation of factors affecting overall hatchling recruitment in this population could be obtained. Furthermore, no eggs were assigned to an ‘unknown’ category during this investigation because by definition, eggs with no evidence of blood vessel or embryo development were categorized as field stage zero. This was based on published research that shows infertility is an unlikely cause of low hatching success in leatherback turtles [25]. Embryonic mortality at each field stage was calculated for each clutch as a percentage of the total number of eggs that died in that clutch. Similarly, the mean number of embryos dying per day of incubation during each developmental stage was computed because stages and incubation duration differed among clutches. Computing the estimated time to complete each developmental stage within a clutch is detailed in the following section.

### 2.3. Precipitation and air temperature

Local historical total daily precipitation and ambient air temperature data from 1990–2010 were provided by the Southeast Regional Climate Centre, obtained from a nearby weather station at Christiansted, St Croix (17°70 N, 64°81 W). Using these data, accumulated precipitation (mm) was calculated for the 30 days immediately preceding the laying date of each nest (representing sand moisture at the time of laying). Additionally, accumulated precipitation and mean temperature (°C) were also determined for the entire incubation period of each nest. The incubation period was defined as the number of days from egg laying to first hatchling emergence. Accumulated precipitation and mean temperature were also calculated from the time of egg laying to the completion of each successive field stage (stages zero–three) in each nest. Estimated time to completion of each developmental stage, expressed as a percentage of total incubation time, has previously been defined for marine turtle embryos [36]. Using this information, it was possible to estimate that from egg laying, it takes approximately 6.7% of incubation to complete field stage zero, 13.4% of incubation to complete field stage one, and 39.4% and 100.0% of incubation to complete field stages two and three respectively. These percentages are cumulative and take into account that to complete field stage one of development, embryos must successfully complete both field stages zero and one, and so on. Applying these percentage values to the known incubation dates for each nest allowed approximation of the date when each field stage finished and the subsequent stage commenced. Accumulated precipitation and mean temperature were then calculated from the time of egg laying to the completion of each successive stage for every nest using these dates.

### 2.4. Data analysis

**Figure 1.**
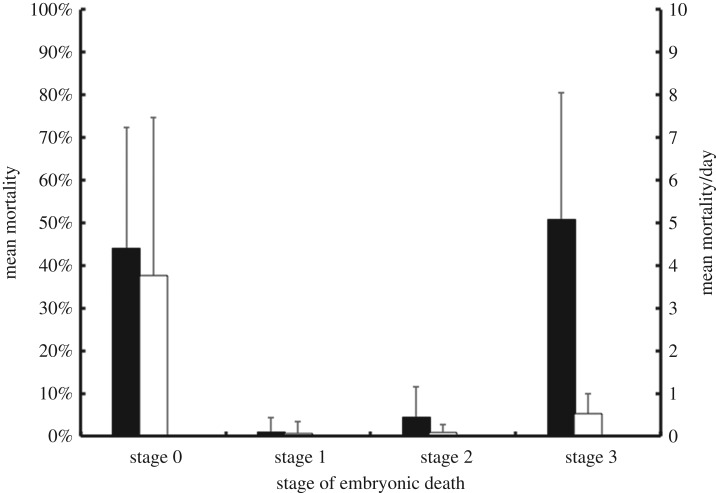
The primary axis (black bars) represents the overall mean embryo mortality (± standard deviation) per developmental stage. The secondary axis (white bars) represents the mean number of embryos dying per day during each developmental stage.

All data were analysed in R, statistical package 3.0 (R Core Team 2013). Test significance was assumed if *p* < 0.05 and verification of model assumptions was conducted prior to analysis. To test how climate variables influenced within nest mortality, linear mixed effects models were constructed using the ‘lme’ function in the ‘nlme’ package. Female ID was treated as the random effect in each model, while hatching success, emergence success and each stage of embryo death (zero–three) were treated as response variables. Accumulated precipitation one month before egg-laying, and accumulated precipitation and mean temperature during incubation were treated as fixed predictor variables. Following preliminary investigations, stages one and two were omitted from subsequent analyses because mean combined mortality was negligible (approx. 5%; figure 1) and the models including these variables had poor explanatory power. Furthermore, collective data for all variables were unavailable during 1991 and 2001 so these years were excluded from analysis. Both *in situ* and relocated nests were included in the analyses.

Models of all possible subsets testing the effects of the three predictor variables on each of the remaining response variables were created using the ‘dredge’ function in the ‘MuMIn’ package. For each model, the maximized log-likelihood (log(*L*)), corrected Akaike's information criterion (AIC_c_), AIC difference (Δ*_i_*), Akaike weight (w_i_) and Nagelkerke *R*^2^ were calculated and models were ranked based on AIC_c_ values. AIC difference (Δ*_i_*) represents the difference in AIC_c_ values between a particular model of interest and the ‘best’ (most parsimonious) explanatory model. Models with Δ*_i_* values ≤2 are considered to have stronger support than those with values ≥2 [37], with the ‘best’ model having the lowest Δ*_i_* value.

To complement model selection, predictor variable importance was established using hierarchical partitioning [38]. This method of analysis identifies the percentage contribution of predictor variables by measuring the goodness-of-fit (log(*L*)) of multiple equivalent models with and without each variable. The percentage total independent contribution of each predictor variable was calculated as a sum across all predictor variables.

Kendall's tau non-parametric correlation test was used to investigate the annual trends in overall mean hatching success, emergence rate, the mean, maximum and minimum air temperature, and mean accumulated precipitation experienced by nests during the primary incubation months (June–August) over the 20 year period, and the relationship of each of these variables to each other.

## 3. Results

### 3.1. Patterns of embryo mortality

A total of 4614 clutches comprising of 357 262 eggs were included in the analyses. There was a significant decline in hatching success (*T *= 47, *p* < 0.01, *τ* = −0.45) and emergence rate (*T* = 45, *p* < 0.01, *τ* = −0.47), both falling from 74% to 55% and from 96% to 91%, respectively. The largest percentage of embryonic death occurred during field stage three (89 038 embryos; 50.62 ± 29.99%), with a mean of approximately 0.50 embryos dying per day per clutch (figure 1). Many embryos also died during field stage zero (71 898 embryos; 44.02 ± 28.42%), although in contrast to field stage three, the mean rate of embryo mortality per day was higher, at approximately 3.8 embryos per day per clutch (figure 1). Embryonic deaths during field stages one and two were lower than field stages zero and three with 1260 embryos (0.86 ± 3.40%) and 6850 embryos (4.21 ± 6.30%) dying at each stage, respectively. The mean rates of embryo mortality at stages one and two were also lower with approximately 0.07 and 0.09 dying per day per clutch, respectively (figure 1).

### 3.2. Climatic influences on within nest mortality

**Figure 2.**
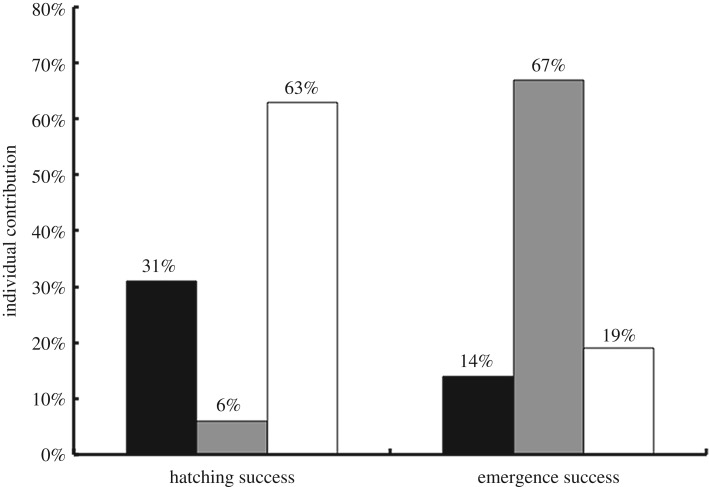
Results of hierarchical partitioning analysis investigating the influence of precipitation and temperature during incubation on clutch hatching success and emergence rate. Black columns represent precipitation before laying (one month), grey columns represent precipitation during incubation and white columns represent temperature during incubation. Values shown are unitless effect sizes and there is no standard accepted method for generating standard errors for these values. Thus no error bars are shown.

Model selection using AIC_c_ resulted in only one model with substantial support (i.e. Δ_*i*_ < 2) for hatching success and three models for emergence rate (table 1). The global model including all three predictor variables (precipitation before laying, precipitation during incubation, and temperature during incubation) accounted for 50% of the variation in hatching success, indicating that collectively they were all important determinants of hatching success within a clutch (table 1). However, further investigation using hierarchical partitioning predominantly supported temperature during incubation as the most important variable to influence clutch hatching success, with an independent explanatory power of 63% (figure 2). An inverse relationship existed between temperature during incubation and hatching success, which decreased within a clutch when temperature increased.

**Table 1.** Linear mixed models testing the impact of precipitation and temperature on clutch hatching success and emergence rate, ranked according to AICc. Only models with strongest support (i.e. Δ*i* ≤ 2) are shown below. The maximized log-likelihood (log(*L*)), corrected Akaike's information criterion (AIC_c_), AIC difference (Δ*_i_*), Akaike weight (w_i_) and *R*^2^ are shown for each model.

	variables							
model	precipitation before laying	precipitation during inc.	temperature during inc.	log(*L*)	AIC_c_	Δ*_i_*	w_i_	*R*^2^
(1) hatching success	X	X	X	3.54	4.9	0.00	1.00	0.50
(1) emergence rate		X		929.62	−1851.2	0.00	0.48	0.39
(2) emergence rate		X	X	930.16	−1850.3	0.94	0.30	0.39
(3) emergence rate	X	X		929.82	−1849.6	1.62	0.22	0.39

With regard to emergence rate, all three models in the set included precipitation during incubation. However, the top-ranked model (lowest AIC value) contained only precipitation during incubation, which explained 39% of the variation in clutch emergence rate (table 1). Hierarchical partitioning also supported the selection of precipitation during incubation as the most influential variable (figure 2). Precipitation during incubation had the highest independent explanatory power of all three variables, which accounted for 67% of the total independent contribution of all variables. Both precipitation before laying and temperature during incubation had smaller, independent contributions of 14% and 19%, respectively. Both statistical methods agree that the variable most likely to influence clutch emergence rate is precipitation during incubation, which significantly strengthens this finding [39]. An overall positive relationship existed between precipitation during incubation and emergence success, with emergence success increasing with precipitation.

**Figure 3.**
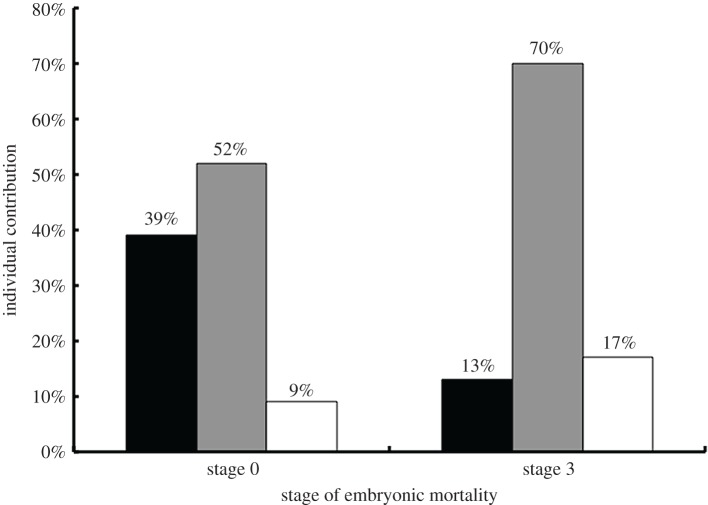
Results of hierarchical partitioning analysis investigating whether precipitation and temperature influence the stage when embryos die during development. Black columns represent precipitation before laying (one month), grey columns represent precipitation during developmental stage and white columns represent temperature during developmental stage. Values shown are unitless effect sizes and there is no standard accepted method for generating standard errors for these values. Thus no error bars are shown.

Model selection using AIC_c_ resulted in two models with substantial support for explaining each of stage zero and stage three mortality (table 2). With regard to stage zero, the top-ranked model included two variables (precipitation before laying and precipitation during developmental stage) and explained 53% of the variation in stage zero mortality. Increased mortality at stage zero was associated with a decrease in precipitation before laying and an increase in precipitation while embryos were at stage zero. In contrast, the top-ranked model for stage three also included precipitation during that developmental stage, as well as temperature during the stage, which explained 53% of the variation in stage three mortality. Increased mortality at stage three was associated with decreased precipitation and increased temperature during the developmental stage. Hierarchical partitioning supported the selection of precipitation during developmental stage as the most important variable influencing both stages zero and three mortality within a clutch, with independent contributions of 52% and 70%, respectively (figure 3).

**Table 2.** Linear mixed models testing the impact of precipitation and temperature on the stage of embryonic death (0 and 3), ranked according to AICc. Only models with strongest support (i.e. Δ_*i*_ ≤ 2) are shown below. The maximized log-likelihood (log(*L*)), corrected Akaike's information criterion (AIC_c_), AIC difference (Δ*_i_*), Akaike weight (w_i_) and *R*^2^ are shown for each model.

	variables							
model	precipitation before laying	precipitation during stage	temperature during stage	log(*L*)	AIC_c_	Δ*_i_*	w_i_	*R*^2^
(1) stage 0 mortality	X	X		−1444.04	2898.1	0.00	0.65	0.53
(2) stage 0 mortality	X	X	X	−1443.67	2899.4	1.27	0.35	0.53
(1) stage 3 mortality		X	X	−1704.81	3419.6	0.00	0.73	0.53
(2) stage 3 mortality	X	X	X	−1704.81	3421.6	2.00	0.27	0.53

### 3.3. Climatic influences on hatchling output

**Figure 4.**
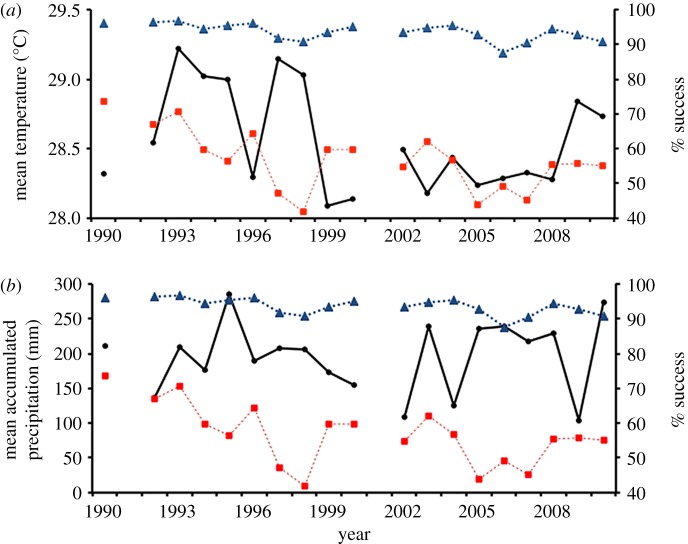
Annual mean hatching success (dashed red line with square) and emergence rate (dashed blue line with triangle) of leatherback nests from 1990 to 2010 (excluding 1991 and 2001) in relation to mean air temperature (*a*) and accumulated precipitation (*b*) during the primary nesting months (June–August).

Mean minimum, maximum and average temperature during the primary nesting months (June–August) were in the ranges 24.7–26.4°C, 30.2–32.4°C and 28.1–29.2°C, respectively, throughout the study period. Total accumulated precipitation during these months ranged from 103 to 285 mm. There were no statistical trends in any of these mentioned climate variables over time, and none were significantly related to overall hatching success or emergence rate (*p *> 0.1). Annual mean temperature and accumulated precipitation experienced by all nests during incubation are shown in figure 4.

## 4. Discussion

Between 1990 and 2010, fluctuations in both temperature and precipitation were associated with an increase in within nest mortality but they were not related to the overall decline in hatchling output at this site. Our results contrast findings at other leatherback nesting sites, where climatic variability driven by the El Niño Southern Oscillation creates a more extreme nesting environment. In northwest Costa Rica, drier and warmer conditions increase leatherback egg and hatchling mortality [9]. Similarly, a decline in hatchling output due to increased temperatures has also been documented in other marine turtle species [40]. In these latter studies, embryo and hatchling mortality occurred when nest temperatures reached or exceeded a lethal threshold, which is generally considered to be 33°C–35°C [9]. Although finite lethal temperature ranges for leatherback turtle embryos are lacking in the literature, the species may be more sensitive to incubation temperature than other species based on a meta-analysis of existing temperature and hatching success data [40]. However, there have been reports of leatherback embryos withstanding short-term exposure to temperatures above 36°C without any detrimental impact on success [41,42].

Ambient air temperature was used as a proxy for sand temperature during this study and is a widely accepted alternative in the absence of direct sand measurements [28,43]. Interestingly, the mean maximum temperatures reported herein (approx. 30–32°C) coincide with data published by Garrett *et al.* that showed maximum within nest temperatures of approximately 31–36°C at this site [31]. The elevated temperature in this latter study was likely due to metabolic heating in the nest chamber, which cannot be captured with ambient air measurements. Binckley *et al.* [41] documented an increase in leatherback turtle nest temperatures of up to 7°C due to metabolic heating, which begins as early as the middle third of incubation. Further, eggs in the centre of a nest experience a greater temperature change and have a lower developmental success than those at the periphery [16,42], although we could not associate egg location with developmental fate in this investigation. Nevertheless, the data reported in this study, and Garrett *et al.* [31], suggest that the temperatures experienced at SPNWR are within the tolerance range for leatherback embryos at this site.

At SPNWR, the overall mean air temperature during incubation from 1990 to 2010 was 28.2°C, which is close to the pivotal temperature for TSD for this species [41]. At these higher temperatures, it is plausible to hypothesize that primary sex ratios may be heavily female biased at SPNWR [44], as is the case at other nesting sites [45]. Historically, the production of more females at higher temperatures provided an evolutionary advantage because it offset rising mortality levels by increasing fecundity [30]. However, there are concerns that a continued reduction in male hatchling output and a subsequent decline or elimination of breeding adult males, as a result of climate change, may have serious detrimental effects on population stability [3,46]. However, recent analysis suggests that female-biased hatchling sex ratios translate into balanced operational sex ratios, until extreme temperatures result in embryonic mortality [47]. Additionally, a recent investigation of sex ratios in the breeding adults at SPNWR revealed 1.02 males for every female, implying that the operational sex ratio is balanced in this breeding stock [48]. The stability of the temperature at this site over the last two decades also suggests that this may be the steady state for this population.

An important finding from this study was the interplay between temperature and precipitation, and how both variables affected each stage of development. To our knowledge this is the first study to document the impact of both climatic variables on the success of embryos in natural nests at each developmental stage. Precipitation had the largest individual impact on the survival of embryos at each stage, but its effects differed markedly during early and late stages of development. Increased precipitation elevated mortality in early stage embryos, whereas it promoted survival of late stage embryos, as well as increased emergence success. Recent work by Wyneken & Lolavar [12] has experimentally demonstrated the importance of nest moisture as a modifier of the effect of temperature on sex determination, with elevated moisture resulting in male turtles being produced from nests that would otherwise be considered female-producing temperatures. The interaction between environmental temperature and environmental precipitation can therefore have important consequences for both survival and sex of embryos.

Increased precipitation at the end of development may promote survival through several mechanisms related mainly to temperature regulation. High temperatures negatively impact survival by decreasing oxygen diffusion in the nest [49]. This phenomenon is particularly relevant during later stages of development when embryonic oxygen demand is highest. Additionally, high nest temperatures also inhibit muscle coordination during hatchling ascent, causing sand desiccation thus making it difficult to ascend, and generally increasing mortality of hatchlings prior to emergence [17,50]. Increased precipitation ameliorates these effects by reducing nest temperature [19,20,51].

High early stage mortality as a result of precipitation is more difficult to interpret. First, it is possible that high precipitation decreases nest temperature below an optimum range for successful growth and development [20]. However, stage zero development following oviposition is independent of temperature in green sea turtles *Chelonia mydas* [52] and so possibly in leatherbacks also. Second, parchment-shelled turtle eggs become turgid from absorbing water vapour in the surrounding nest environment after oviposition, but it is actually subsequent water loss that appears to be more detrimental to development [15]. Finally, decreased diffusion of oxygen throughout the nest as a result of precipitation may negatively impact development [21]. Although oxygen consumption of early embryos is negligible, oxygen is the trigger necessary to break preovipositional arrest of embryos following oviposition [53,54]. Although a threshold concentration of oxygen required by embryos to break arrest has not been established, it is possible that oxygen levels in the nest following precipitation are too low to facilitate this, resulting in embryo mortality.

Interestingly, the patterns of mortality seen at SPNWR are somewhat different from those reported for other leatherback populations. The embryos of leatherbacks nesting in the Pacific primarily die during stage zero, presumably because they fail to recommence development at laying following a period of preovipositional arrest *in utero* [25,27]. In contrast, in our investigation, stage three mortality accounted for the largest percentage of overall clutch mortality. However, taking the number of embryos dying per day into account, stage zero remains the most significant stage in influencing clutch success. Nevertheless, the overall effects of increasing embryo mortality are likely to impact the stability of the SPNWR population in 10–15 years' time when hatchlings reach maturity. At such a time, the existing increasing trend in population growth [32] will likely be reversed [55].

In conclusion, our findings suggest that despite influencing within nest mortality, climatic variability does not account for the overall decrease in hatchling output at SPNWR from 1990 to 2010. Further research is therefore needed to elicit the reasons for this regional decline. Continued monitoring of hatchling output and observation of trends in climate variables at this site will assist in early identification of potentially detrimental changes if they arise in the future.

## Data accessibility

Our data are deposited at Dryad: http://dx.doi.org/10.5061/dryad.889h8 [56].
